# Supplementary material for: Chromothripsis during telomere crisis is independent of NHEJ, and consistent with a replicative origin
Source: Genome Res. 2019 May;29(5):737–49. doi: 10.1101/gr.240705.118 (PMC6499312; doi:10.1101/gr.240705.118)
Supplement: Supplemental Material [file supp_gr.240705.118_Supplemental_file_1.zip › contigs/annotated_contigs/DB82/contig.2.DB82_length_418_mean_cov_7.89473684211.docx]

**DB82_length_418_mean_cov_7.89473684211**

```
AAGAGAAAGGGTAAGGTGAGTTCCGGTTTATGATTTGACTTGAGGCATTCGGAGGCTAAACATCCACAAGGTTAGTTCCCTGAACCCCT
 >chr20:8286291-8286558 + E=3e-149
ATAAACCTTGCTATTTATATCTCTTCCTCAACTCTGAGGCCCTCACCCTACATCGGCTGCAGTACAGATTCAGCCTTTAGCGACGGAAT

TATGTACTGAAACACTCATTTCTGTGCCGCTGCAGTGGTTAGCTTTGATTTTTATAGAGATGCACATGAGAAAACAGTCTGAGC|CTGG
                                                                                     >ch
A|TTCAGCTTTAACTGGTTCTAAAATAATGAAGCAGAAATGACTTTCCTTCTCTCTCTGGTGTCAGCTTCAAATGAATTATGGATACTG
r20:8287918-8288074 + E=2e-82
AACCTACAGAGGTAATGCTGATTAAATAATTGAACAATCCATTGATTAACTACAGATTGTCAAT
```
